# SimVec: predicting polypharmacy side effects for new drugs

**DOI:** 10.1186/s13321-022-00632-5

**Published:** 2022-07-26

**Authors:** Nina Lukashina, Elena Kartysheva, Ola Spjuth, Elizaveta Virko, Aleksei Shpilman

**Affiliations:** 1 AI Labs, JetBrains Research, Saint-Petersburg, Russia; 2 Center for Data Analysis and Machine Learning, HSE University, Saint-Petersburg, Russia; 3 Department of Pharmaceutical Biosciences and Science for Life Laboratory, Uppsala University, Uppsala, Sweden; 4 Data Analytics Team, JetBrains, Saint-Petersburg, Russia

**Keywords:** Polypharmacy, Knowledge graph

## Abstract

Polypharmacy refers to the administration of multiple drugs on a daily basis. It has demonstrated effectiveness in treating many complex diseases , but it has a higher risk of adverse drug reactions. Hence, the prediction of polypharmacy side effects is an essential step in drug testing, especially for new drugs. This paper shows that the current knowledge graph (KG) based state-of-the-art approach to polypharmacy side effect prediction does not work well for new drugs, as they have a low number of known connections in the KG. We propose a new method , SimVec, that solves this problem by enhancing the KG structure with a structure-aware node initialization and weighted drug similarity edges. We also devise a new 3-step learning process, which iteratively updates node embeddings related to side effects edges, similarity edges, and drugs with limited knowledge. Our model significantly outperforms existing KG-based models. Additionally, we examine the problem of negative relations generation and show that the cache-based approach works best for polypharmacy tasks.

## Introduction

### Polypharmacy side effects prediction

Polypharmacy is commonly defined as the use of drug combinations on a daily basis, which may be good practice for the treatment of many complex and terminal diseases. Although this strategy might often be efficient [1], patients have a higher risk of side effects due to drug-drug interactions [2]. It is practically impossible to test all possible pairs of drugs and side effects are usually not observed in relatively small clinical testing [1]. Therefore, a reliable automated prediction of polypharmacy side effects is an essential task for the healthcare industry.

A traditional way to identify effective drug combinations is an experimental screening of all possible combinations in a predefined set of drugs [3]. A computational prediction of the interacting drug-drug pairs could accelerate the process of screening possible candidates. Drug-drug interaction prediction approaches developed before 2018 can be categorised [4] into classification-based [5–7] and similarity-based methods [8, 9].

Both classification-based and similarity-based approaches predict a scalar value that represents the overall strength of the interaction for a given drug pair. Still, they cannot predict the exact type of side effect. To overcome this limitation, the Decagon model based on knowledge graphs was developed in 2018 [4]. This model predicts the exact side effect between a specified pair of drugs by solving a multi-relational link prediction task in multimodal networks. The model acts in a manner of an encoder-decoder framework with a GCN-based encoder and tensor factorization decoder.

Following a surge in research on graph representation learning in recent years, [10] suggested using other graph embedding models, such as TransE, DistMult, and KBLRN. Recently, the latest TriVec model [11] uses a tensor-factorization-based knowledge graph embedding model that extends the work of the DistMult and ComplEx models.

### Knowledge graphs

A concept of a knowledge graph (KG) has no single commonly accepted definition [12] . Historically, KGs were formulated as a special case of semantic networks, with additional constraints to facilitate algebras on a graph [13]. KGs are widely used in search engines, natural language processing, and semantic reasoning.

In a context of graph representation learning, KGs are usually defined as a network of real-world objects, whose nodes represent entities of interest and whose edges represent potentially different relations between these entities. Notably, nodes and edges could be of different types, standing for different kinds of real-world objects and relations between them. The types of objects and relations, along with other properties of the nodes and edges, are represented in a KG with node labels and edge labels, respectively. Given such a graph, one can formulate one of the two main tasks on a subgraph level:

- Node classification: predict a label for a node
- Link prediction: predict if an edge (of a particular type) exists between a pair of nodes

A detailed description of tasks in graph machine learning is beyond the scope of this paper; for more information, we refer the reader to [14].

In order to solve these tasks, machine learning algorithms use graph structure and labels to aggregate information and represent a graph via low-dimension vectors. Thus, by varying the structure of a graph or corresponding labels, one can modify the prediction results.

For the prediction of polypharmacy side effects, Decagon’s [4] authors constructed the first KG and formulated the polypharmacy side effects prediction problem as a link prediction problem in a heterogeneous graph. The constructed KG has been widely used in later research. The nodes in this graph represent drugs and proteins, while the edges represent drug-drug and protein-protein interactions. We discuss the polypharmacy KG in more detail in the Materials and Methods section.

### Limitations of KG-based models

Unlike classification-based and similarity-based methods, KG-based methods for polypharmacy prediction do not use information on the chemical structure of drugs and their biological activity. KG-based models rely only on the graph of facts without any additional assumptions. Therefore, if there is a small number of known facts for a particular drug, the model will have difficulty predicting new links. This could be a serious problem for the practical use of the prediction system due to the fact that new drugs are in the most need of analysis, while they do not have many known polypharmacy assertions. Therefore, the addition of learning embeddings of drugs according to their chemical structure allows the learning of polypharmacy side effects of drug combinations with a low number of known connections in the knowledge graph [11].

The main contribution of this paper is an improvement in prediction of polypharmacy side effects for drugs with limited known polypharmacy assertions. We enhance the knowledge graph structure by taking chemical structure into account upon node initialization and a new type of edges corresponding to the chemical similarity between drug nodes. These edges are weighted and aim to propagate information about side effects between similar drugs. We introduce a 3-step process that learns the weak node embeddings using the information about single side effects. Finally, our model incorporates a cache-based process of generating negative relations.

**Table 1:** Results of KG-based models

Model	Uniform split		Weak nodes split	
	ROC AUC	AUC PR	ROC AUC	AUC PR
RESCAL_original_a	0.693	0.613	0.44 ± 0.015	0.535 ± 0.08
RESCAL_chem_	0.693 ± 0.001	0.613 ± 0.002	0.374 ± 0.003	0.479 ± 0.004
RESCAL_SE_	0.693 ± 0.001	0.613 ± 0.001	0.371 ± 0.002	0.469 ± 0.002
Decagon_original_SE_a	0.872	0.832	0.3 ± 0.02	0.45 ± 0.008
Decagon_chem_	0.821 ± 0.005	0.785 ± 0.004	0.321 ± 0.003	0.445 ± 0.004
TriVec_original_a	0.975	0.966	0.44 ± 0.016	0.535 ± 0.07
TriVec_SE_	0.975 ± 0.002	0.967 ± 0.002	0.397 ± 0.002	0.492 ± 0.003
SimVec_chem_	0.975 ± 0.002	0.966 ± 0.003	0.513 ± 0.009	0.568 ± 0.004
SimVec_SE_	0.975 ± 0.003	0.967 ± 0.002	0.742 ± 0.006	0.719 ± 0.005
SimVec_weighted_	0.975 ± 0.001	0.967 ± 0.001	0.525 ± 0.008	0.571 ± 0.005
SimVecVec_chem_weighted_	0.975 ± 0.002	0.966 ± 0.003	0.51 ± 0.006	0.56 ± 0.003
SimVec_SE_chem_	0.975 ± 0.001	0.967 ± 0.002	0.741 ± 0.007	0.719 ± 0.005
SimVec_SE_weighted_	0.975 ± 0.002	0.967 ± 0.002	0.764 ± 0.005	0.739 ± 0.004
SimVec_full_	0.975 ± 0.002	0.968 ± 0.003	0.773 ± 0.006	0.745 ± 0.003

^a^Reported in [11]

**Table 2:** False positive rate and false negative rate for different versions of SimVec model

Model	FPR	FNR
SimVec_chem_	0.46	0.52
SimVec_SE_	0.26	0.16
SimVec_weighted_	0.47	0.43
SimVec_chem_weighted_	0.45	0.53
SimVec_SE_chem_	0.37	0.13
SimVec_SE_weighted_	0.26	0.14
SimVec_full_	0.25	0.11

**Table 3:** Results of negative sampling sampling models on weak nodes split

Model	ROC AUC	AUC PR
Uniform_6:1_	0.753 ± 0.005	0.730 ± 0.003
Bernoulli	0.752 ± 0.007	0.723 ± 0.005
NSCaching	0.763 ± 0.004	0.736 ± 0.005
Exploration	0.778 ± 0.006	0.748 ± 0.005
NSCaching_6:1_	0.780 ± 0.006	0.750 ± 0.005
Strong NSCaching	0.785 ± 0.006	0.755 ± 0.005
Stay Positive	0.849 ± 0.007	0.827 ± 0.007

**Table 4:** Computational resources for training KG-based models on weak nodes split

Model	Time (min)	GPU (GB)	CPU (GB)
RESCAL_original_	153	5.4	3.9
RESCAL_chem_	153	5.4	3.9
RESCAL_SE_	153	5.4	3.9
Decagon_original_SE_	13254	11	9.5
Decagon_chem_	12877	9.6	7.8
TriVec_original_	101	1.3	3.9
TriVec_SE_	102	1.4	3.9
SimVec_chem_	102	1.4	3.9
SimVec_SE_	112	1.5	4.1
SimVec_weighted_	118	1.7	4.2
SimVecVec_chem_weighted_	119	1.7	4.2
SimVec_SE_chem_	114	1.6	4.2
SimVec_SE_weighted_	128	2	4.5
SimVec_full_	128	2	4.5

**Table 5:** Training time of different version of SimVec model on a server without GPU acceleration

Model	Time (min)
SimVec_chem_	1460
SimVec_weighted_	1757
SimVec_SE_	1612
SimVec_chem_weighted_	1761
SimVec_SE_chem_	1614
SimVec_SE_weighted_	1947
SimVec_full_	1948

## Methods

### Dataset

In this study, we use a preprocessed dataset, provided within the Decagon paper [4]. The dataset incorporates human protein-protein interaction networks, side effect data, and drug-target interactions, all compiled from different sources.

Decagon’s [4] authors use these data to construct a knowledge graph (KG). The graph consists of nodes of two types, drugs and proteins. The edges of the graph correspond to different protein-protein interactions, drug-protein interactions, and drug-drug interactions. Each drug-drug interaction is labeled by a different edge type, which signifies the type of side effect. The edges of the graph are then divided into train, test, and validate sets.

### Problem definition

All the state-of-the-art KG-based polypharmacy side effects prediction models (such as RESCAL [15], KBLRN [10], Decagon [4], TriVec [11], etc.) use the described knowledge graph. In these models, the learning of the embeddings is done through the propagation of information through the edges during training. If a drug has a limited number of known polypharmacy assertions, its embedding vector will not change much. In the extreme case of an isolated node, it will not change at all. The model will not be able to produce meaningful predictions for such drugs. Notably, in practice, such drugs most closely correspond to new drugs, which are especially important to be tested against possible polypharmacy side effects. To illustrate this problem, we emulate the practical testing of new drugs, by using new train/validate/test split, such that new drugs occur only in test and validation triples, while training dataset consists of other better-studied drugs. This new split is referred to as a ”weak nodes split” later in this paper.

### Weak nodes split

The weak nodes split is created in the following manner .

In polypharmacy KG , new drugs correspond to the nodes with a low degree due to the fact that such drugs usually do not have many known polypharmacy assertions . We denote new drugs of interest as a set of nodes, consisting of *N* nodes with the smallest number of existing edges in the polypharmacy KG. This set is obtained by sorting the nodes in ascending order by their degree and getting first *N* of them.

The set of *N* nodes (new drugs) has a corresponding set of *M* triples, which have at least one of its incident vertices being a new drug. We later denote such triples as ”weak triples”. We put *M*/2 of weak triples in the test dataset, other *M*/2 of weak triples in the validation dataset and the rest of the triples present in KG in the training dataset. Thus, we construct a new train/validate/test split which aims to test models’ performance on newly developed drugs.

Parameters *N* and *M* could be varied taking into account the following considerations. The number of weak triples *M* is directly related to the number of weak drugs *N*; as one of these parameters increases, so does another. The number of weak triples *M* is, in fact, a size of the validation and testing datasets. The larger is a size of a testing dataset, the more reliable is evaluation result. Therefore, the largest possible value of *M* is desirable. However, with an increase of *N* more drugs are considered new drugs, and the size and diversity of the training dataset decrease. Therefore, there is a trade-off between *M* and *N*. In our experiments, we use $N = 98$ (with corresponding $M = 20000$), which is approximately 1/6 of the number of drugs in KG, as it is the smallest size of the set of new drugs having stable results during our experiments.

**Fig. 1:**
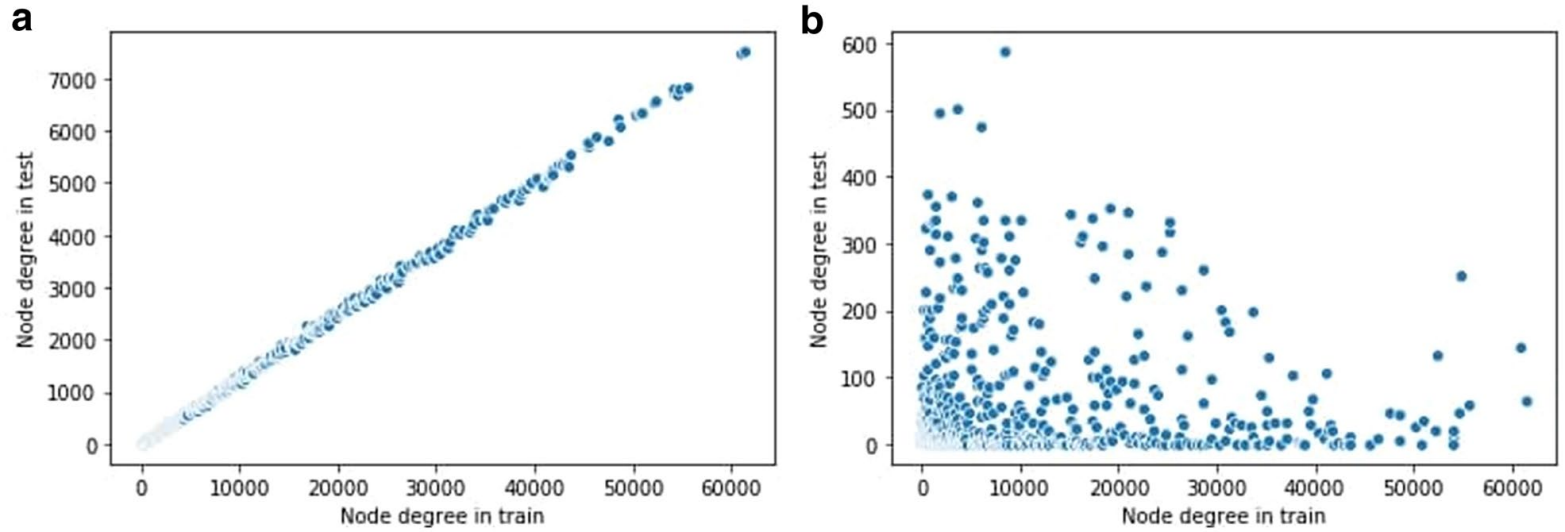
Distribution of node powers in train and test sets.** a** In uniform split each node preserves its degree both in train and tests data sets. That is, for each node there are approximately equal number of train and test triples.** b** In weak nodes split there are many nodes, which have small number of train triples and high number of test triples. Such nodes have low degree in train and high degree and test, and emulate testing of new drugs

Figure 1 shows the changes in the distribution of node powers in the test set after applying the procedure mentioned above. While the original uniform split produces an extremely small percentage of weak nodes, the new split contains a large number of weak edges alongside some of the strong edges.

### Performance of the existing models on the weak nodes split

We evaluated three KG-based models on this split. The results, listed in Table 1, show that these models do not work well in this scenario. We report the averaged ROC AUC and AUC PR scores, as reported in [4]. During the evaluation, for each possible relation *r*, a model tries to predict if each triple $(h, r, t)_{test}$ from the test set exists or not. The final score is an average over all possible relations.

We can explain the low scores of the current state-of-the-art TriVec model as follows. At first , each drug node in the graph is initialized with a random vector close to zero. During training, for each triple $(h, r, t)_{train}$ in the training set, the embeddings of nodes *h* and *t* and relation *r* are updated. However, for new unknown drugs , their node embeddings are not updated because they do not have known relations in the training set. Thus, after training, weak nodes in the graph have random embeddings, which differ significantly from the learned ones.

Consequently, in terms of the trained model, a triple $(h, r, t)_{test}$ with a weak node *h* is similar to a triple $(h', r, t)_{train}$, where $h'$ is a node with an embedding close to a random vector. During testing, a test triple $(h, r, t)_{test}$ with weak node *h* produces a biased prediction, similar to a prediction of $(h', r, t)_{train}$.

In this paper, we propose a new model, SimVec, which works significantly better for new drugs.

### SimVec model

#### Enhanced knowledge graph

**Fig. 2:**
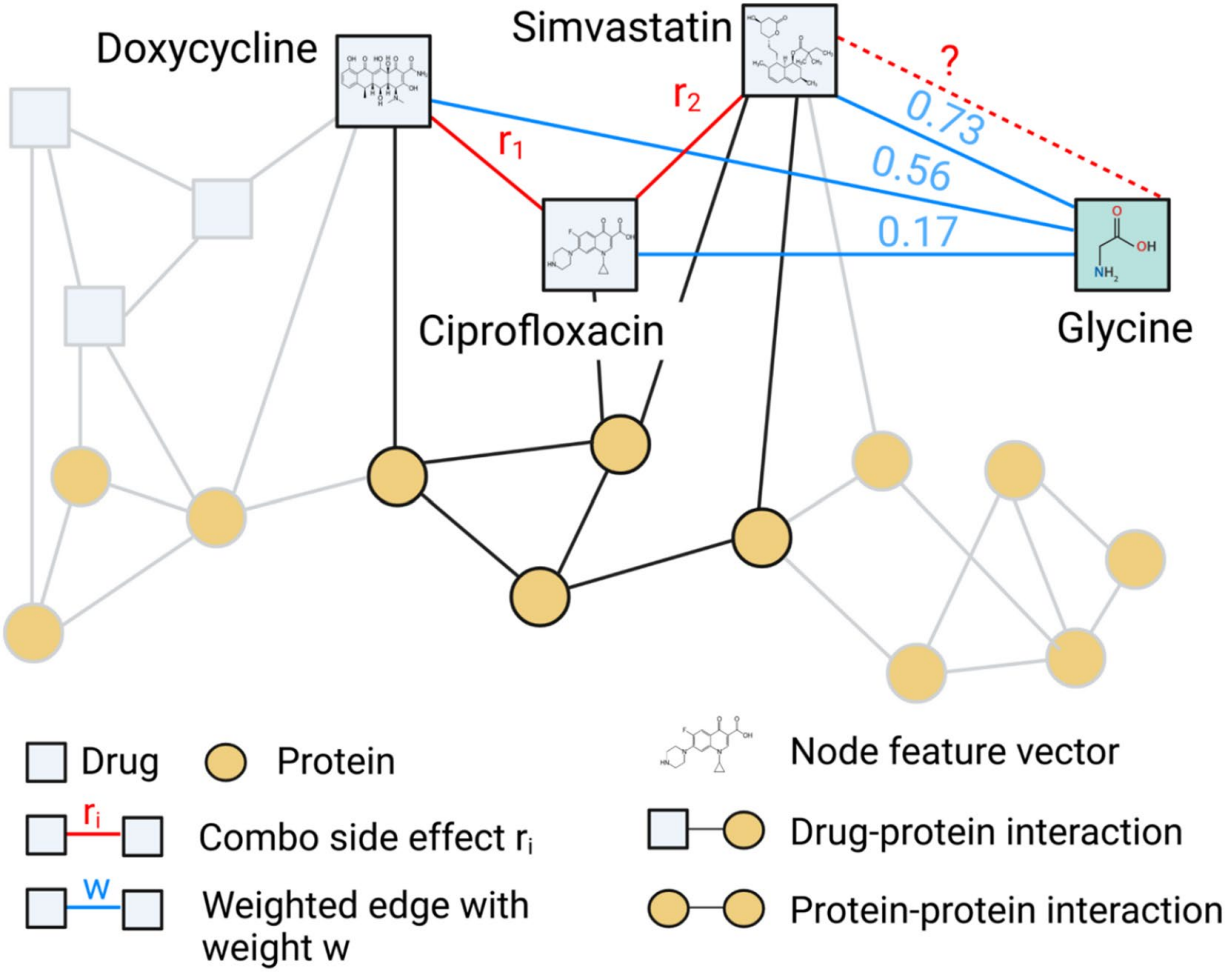
SimVec KG, a multimodal graph, which contains 645 drug and 19081 protein nodes. Each node is considered being a molecule, represented by an embedding. Graph edges correspond to protein-protein interactions, drug-protein targets, and drug-drug side effects interactions. In addition, weighted drug-drug similarity edges connect drugs with respect to their similarity

We enhance this knowledge graph with structure-aware node initialization and weighted drug-similarity edges (Fig. 2).

As previously, there are two types of nodes (entities): drugs and proteins. Following the idea of TriVec’s tensor factorization-based knowledge graph embedding model [11], we represent each entity *i* using three embedding vectors of the same size: $\Theta _E(i) = \{e_i^1, e_i^2, e_i^3\}$. We consider each node as a molecule, and thus its embedding is represented by a chemical embedding. We propose several alternatives to the choice of these chemical embeddings and introduce the corresponding drug similarity metrics.

The new graph preserves protein-protein interactions, drug-protein interactions, and drug-drug interactions (side effects edges), as well as a new type of weighted drug-drug edges. These edges link each drug to the others, and the weight of the edge corresponds to the chemical similarity between the drugs. Similarly to the nodes, we represent each relation *j* with $\Theta _R(j) = \{w_j^1, w_j^2, w_j^3\}$. Each embedding of a relation is randomly initialized with the Xavier initialization [16].

#### Node initialization

We propose to initialize drug nodes in the knowledge graph with chemical embeddings. The intention is to give the model some prior knowledge about the chemical structure of the drugs to improve the reasoning for the weak nodes. If there is a limited number of known polypharmacy side effects for a particular drug, the model could make assumptions based on the drug’s chemical properties. In our study, we used three types of 100-dimensional chemical embeddings, including circular fingerprints [17], molecular descriptors as vectors of global molecular features, and embeddings from pretrained hyperbolic VAE [18]. We also propose different alternatives for the similarity metric *s*(*X*, *Y*) of the embeddings *X* and *Y*.

##### Circular fingerprints

The use of circular fingerprints is a standard approach to molecular representations, with the corresponding similarity metric of Tanimoto score: $similarity(X, Y) = \frac{\sum _i (X_i \wedge Y_i)}{\sum _i (X_i \vee Y_i)}$.

In our implementation, the circular (Morgan) fingerprints are generated based on the canonical SMILES [19] representation of each node. We obtain the canonical SMILES through the PubChem service [20]. We then generate circular (Morgan) fingerprints using the RDKit package [21]. We use a radius of the fingerprint equal to 3, as one of the common choices.

##### Molecular descriptors

Molecular descriptors are vectors of the chemical properties of a molecule. There is no common similarity metric for such property-based molecular descriptors; we suggest using the Gaussian kernel: $similarity(X, Y) = exp(-\frac{|| X - Y ||^2}{\sigma ^2})$, where $\sigma$ parameter can be varied.

In our implementation, we use 191 molecular descriptors available from the RDKit package [21]. Since the selected dimension of the embedding is 100, we select the properties with the highest number of unique values for our dataset. The resulting list of descriptors is provided in the Appendix of the manuscript.

##### Hyperbolic VAE

Hyperbolic VAE is one of the modern approaches to representation learning, which naturally leads to the similarity metric of hyperbolic embeddings in hyperbolic space: $similarity(X, Y) = \frac{max_{i, j}(lor\_dist(i, j)) - lor\_dist(X, Y)}{max_{i, j}(lor\_dist(i, j))}$, where $lor\_dist(X, Y) = -X_0Y_0 + \sum _{i=1}^n X_iY_i$.

In our implementation, we use the implementation provided by the authors of the method [18]. To train the model, we use all hyperparameters with the default values mentioned in the project GitHub (https://github.com/pfnet-research/hyperbolic_wrapped_distribution), except the latent space size , which we set to 100 for node embeddings.

#### Weighted similarity edges

We introduce a new type of weighted edges that correspond to the chemical similarity of the drugs. Each drug node in the graph is linked to each of the remaining drug nodes with a weight that represents how similar the nodes are in terms of chemistry.

We propose two ways to weight such edges. The general idea is to make the weight directly proportional to the similarity score and inversely proportional to the degrees of each incidental vertex. Thus , nodes with a lower number of existing edges will rely more on the information from chemically similar drugs.

The first way of weighting is:

1 $$\begin{aligned} weight(h, t) = similarity(h, t) InvDeg(h) InvDeg(t), \end{aligned}$$

where *InvDeg*(*x*) is an inverse proportion of the normalized vertex degree:

2 $$\begin{aligned} InvDeg(x) = 1 - \frac{deg_x - min_i(deg_i)}{max_i(deg_i) - min_i(deg_i)}, \end{aligned}$$

where $deg_i$ if the degree of node *i*, *i* goes through all drug nodes in the graph.

The second way of weighting is:

3 $$\begin{aligned} weight(h, t) = exp\left(-\frac{||h - t|| ^ 2}{window\_size^2(h, t)}\right) \end{aligned}$$

where $window\_size(h, t) = l\_bound + InvDeg(h) InvDeg(t) (u\_bound - l\_bound)$, and the size of the window is varied from $l\_bound$ to $u\_bound$.

### SimVec learning

We introduce a 3-step training process that iteratively updates the embeddings of (1) incident verities of side effects edges, (2) incident verities of weighted similarity edges, and (3) weak drug nodes. At each learning step during the epoch, the embeddings are updated by minimizing the following loss functions:

1. For side effects edges, we use the loss function, proposed in [11],
  4 $$\begin{aligned} {\begin{matrix} L_{spo}^{TriVec} = -\phi _{spo} + log(\sum _{o'}exp(\phi _{spo'})) -\phi _{spo} + log(\sum _{s'}exp(\phi _{s'po})) \\ + \frac{\lambda }{3}\sum _{k=1}^K \sum _{m=1}^3 (|e_s^m|^3 + |w_p^m|^3 + |e_o^m|^3), \end{matrix}} \end{aligned}$$
  where *K* denotes the embedding size, $\phi _{spo}$ denotes the score of the (*s*, *p*, *o*) triple, $x'$ represents an entity $e: e \ne x, e \in E$, $e_i^m$ is the embedding part *m* of the entity embedding $\Theta _E(i)$, $w_i^m$ is the embedding part m of the relation embedding $\Theta _R(i)$, *m* denotes the embedding part index, $\lambda$ denotes a configurable regularization weight parameter, and |*x*| is the absolute of *x*. Scores $\phi _{spo}$ are computed as follows [11]:
  5 $$\begin{aligned} \phi _{spo} = \sum ^K e_s^1w_p^1e_o^3 + e_s^2w_p^2e_o^2 + e_s^3w_p^3e_o^1, \end{aligned}$$
  where *K* is the length of the embedding vectors. In our work, we use $K = 100$.
2. For weighted similarity edges, we suggest using a new loss function $L_{spo}^{SimVec} = weight(h, t)*L_{spo}^{TriVec}(h, r_{weighted}, t)$.
3. Inspired by the Decagon model, we suggest that drugs with limited known connections should have embeddings close to the embeddings of the drugs, which are similar in terms of single side effects. For each weak drug, we find the top *N* closest drugs in terms of the number of common single side effects. We then encourage the model to learn embeddings by minimizing the MSE loss function between weak and strong nodes. In our experiments we vary *N* from 2 to 20 and pick the best option in terms of the highest ROC AUC value on the validation set from 10 runs, $N = 6$. This hyperparameter *N* could be seen as a trade-off between learning the embedding with respect to the most similar drug in terms of single side effects and allowing the embedding to be the average of a large number of similar drugs.

### Negative sampling strategies

The knowledge graph only has positive examples, which are known facts about polypharmacy assertions. To get a set of negative examples, it is common practice to generate them using a negative sampling strategy. All KG-based models under consideration use the following way to generate negative facts. Given a positive example in the form of an existing edge in KG, negative facts can be generated by replacing one of the two linked vertices with a uniformly sampled random vertex from KG [22].

This process of negative sampling can produce false negatives, which can reduce the performance of the model [23]. Notably, for drugs with a small number of known connections, such false negatives are much more probable because many assertions are missing in KG and are treated as negatives. There is also a trade-off between accuracy and training time , depending on the number of negative examples generated per positive example [24]. In this paper, we investigate the following alternatives to uniform negative sampling.

#### Bernoulli sampling

Bernoulli sampling [25] tries to reduce the number of false negative examples by changing the equal probabilities of replacing a head or a tail of the edge with the probabilities depending on the relation cardinality.

#### Stay positive

Stay Positive model [26] proposes a novel regularization term to the loss function, which obviates the need for negative sampling.

NSCaching. The cache-based NSCaching model [23] keeps track of rare negative triplets with large scores by storing them in a cache. Negative examples are sampled from the cache with a ratio of 1 negative example per positive triple. NSCaching method has a parameter $N_{random}$, which is used in the cache update procedure and can be varied to balance exploration and exploitation.

#### NSCaching_6:1_

Following the idea that an increase in the number of negative examples could improve the accuracy of the model [24], we examine the NSCaching model with an increase in the number of negative samples per positive, specifically, 6–1.

#### Exploration

 We also consider an increase in the number of negative examples per positive as a special case of exploration. Consequently, we hypothesise that it could be useful to make the negative examples even more diverse. To do so, we doubled the size $N_{random}$ of random set in a cache update procedure.

#### Strong NSCaching

**Fig. 3:**
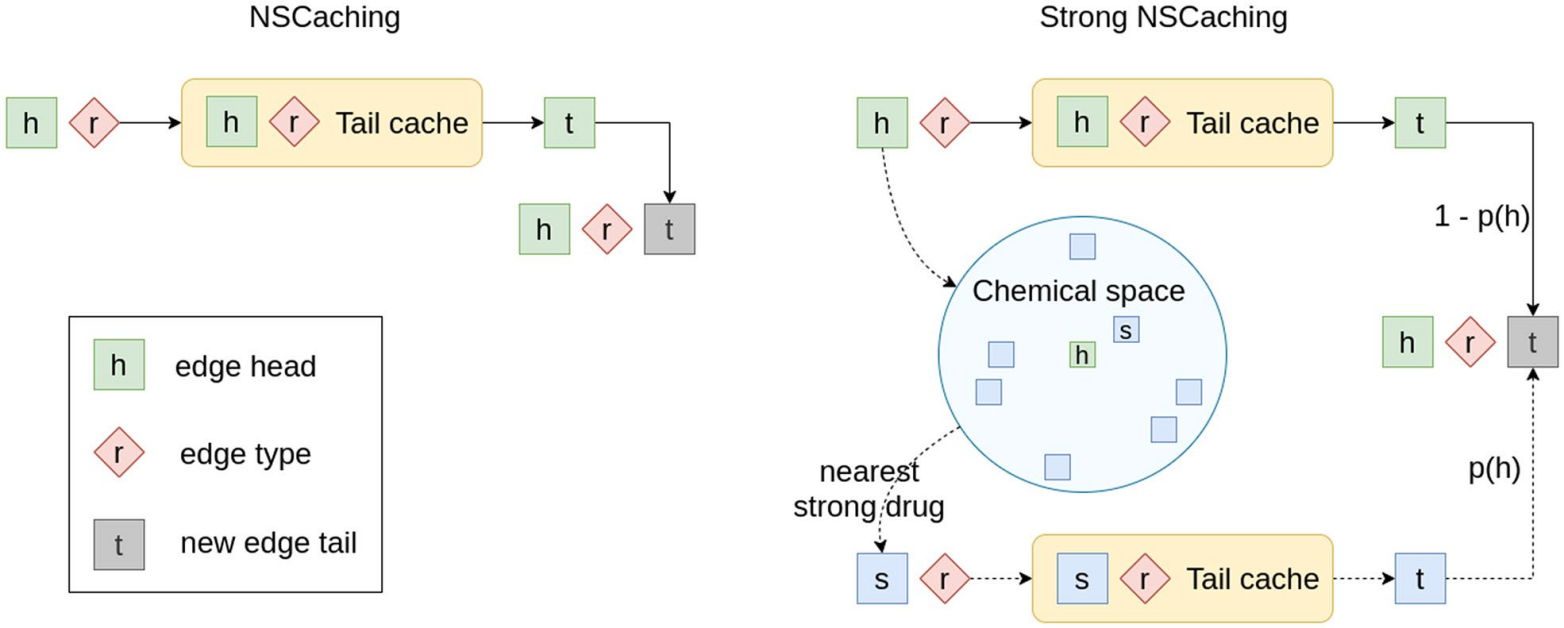
The architecture of NSCaching and Strong NSCaching methods. In NSCaching, the negative triples for a head node *h* and relation *r* are sampled from their tail cache. In Strong NSCaching, with a probability *p*(*h*), which depends on the *h* degree, negative examples are sampled from the cache of the *h*’s nearest strong neigbor in chemical space

We propose a new method, Strong NSCaching, shown in Fig. 3.

We suggest that , for a weak node, it might be effective to use some examples of its strong neighbor. We hypothesize that this intention will work due to the fact that strong edges appear more frequently during training, and the cache for strong nodes is updated more frequently, which leads to better examples in cache for strong nodes.

Strong NSCaching samples from the cache of the nearest strong neighbor with a probability

6 $$\begin{aligned} p(h) = \frac{1}{log(deg_h)^\alpha }, \end{aligned}$$

and sample from the node’s cache with a probability of $1-p$. Parameter $\alpha$ aims to balance probability *p*(*h*) decay rate. The higher is the $\alpha$, the lower is the probability of sampling from the cache of the nearest strong neighbour. Therefore, with large values of $\alpha$, the probability *p*(*h*) becomes close to 0 and Strong NSCaching method becomes similar to the NSCaching method.

In our implementation, to find the nearest neighbor of a node, we use molecular descriptors , as they show the best performance in terms of edge weighting (see Results and discussion section). We check $\alpha = \{\frac{1}{3}, \frac{2}{3}, 1\}$ and pick up $\alpha = \frac{1}{3}$.

## Results and discussion

### Evaluation procedure

In our experiments , we train models with a learning rate $\lambda =0.001$, using an early stopping strategy with $epsilon=0.001$ and $k=7$. TriVec is the only model trained with another learning rate. We found that the value of the learning rate $\lambda =0.005$ provides the best results for this model.

We run all models 10 times on a server with one Tesla k80 accelerator, 61 GiB memory , and 4 virtual CPUs. The source code is available at https://github.com/jbr-ai-labs/simvec. We report the mean ROC AUC and AUC RP scores with standard deviations.

### Performance results

Table 1 shows the evaluation results of the following proposed models with uniform negative sampling:

- RESCAL_original_ is a model from [15]. RESCAL_chem_ and RESCAL_SE_ are the RESCAL models in which drug nodes are initialized with chemical embeddings and single side effects respectively.
- Decagon_original_SE_ is the Decagon model [4]. Notably, Decagon model uses node initialization with one-hot predefined single side effects. Decagon_chem_ is the Decagon model, where nodes are initialized with chemical embeddings instead of single side effects.
- TriVec_original_ is the model from [11]. TriVec_SE_ is the TriVec model, in which drug nodes are initialized with single side effects.
- SimVec_chem_ is the model initialized with chemical embeddings.
- SimVec_weighted_ is the model with weighting and new similarity edges.
- SimVec_SE_ is the model with single side effects based loss function.
- SimVec_chem_weighted_ encapsulates both chemical initialization and weighting.
- SimVec_SE_weighted_ corresponds to both single side effectsbase loss function and weighting.
- SimVec_full_ encapsulates all the changes, namely chemical initialization, weighting, and the updated learning process.

Table 1 shows that all SimVec models produce better results than other KG-based methods on weak nodes split . The performance of all SimVec models on uniform split remains at the same top level as that of the previous methods. Among all proposed modifications, SimVec_full model was proved to have the best results.

**Fig. 4:**
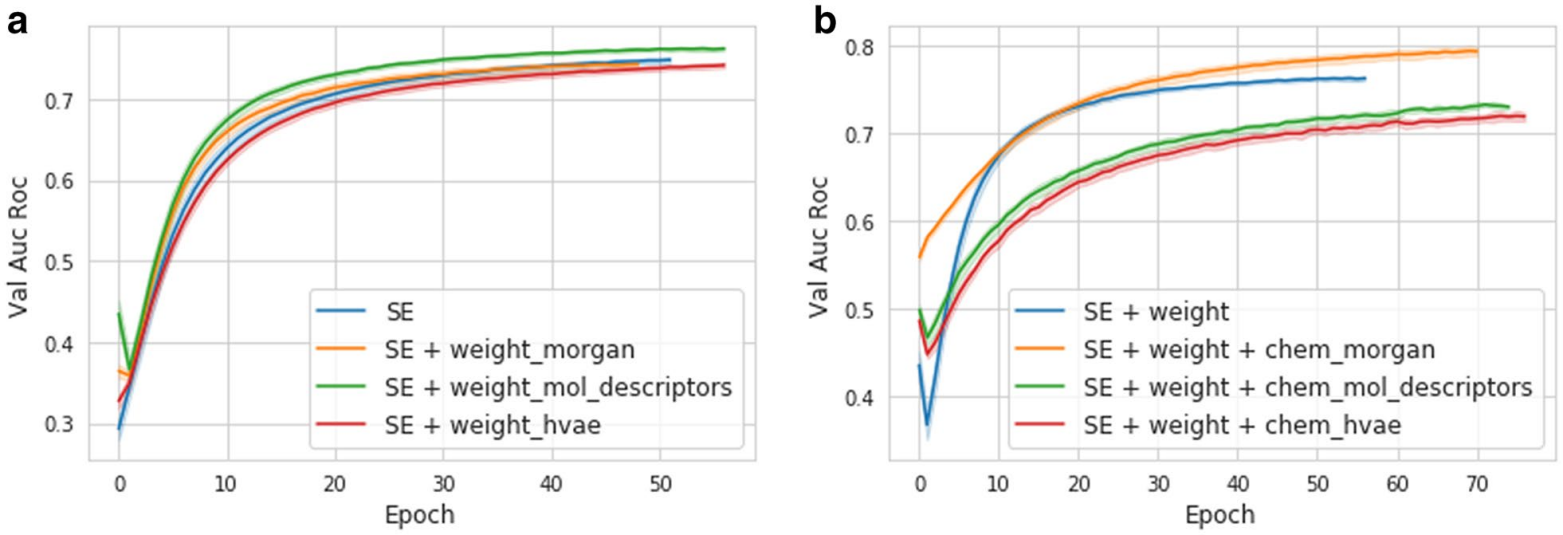
The ROC AUC curves on the validation dataset during the training of SimVec modifications with different (**a**) similarity weighting strategies and (**b**) chemical initialization options. **a** Three different weighting strategies were examined: proportional weighting with similarity functions based on morgan fingerprints (weight_morgan) and hyperbolic embeddings (weight_hvae), and window weighting based on molecular descriptors (weight_mol_descriptors). Albeit all the strategies show similar performance, molecular descriptors with window weighting work best. **b** Among three available chemical initialization options, namely, morgan fingerprints (chem_morgan), RDKit-based molecular descriptors (chem_mol_descriptors) and hyperbolic embeddings (chem_hvae), morgan fingerprint work best

Since we proposed different ways of structure-aware node initialization and ways of edge weighting, we conducted experiments to choose the best options. Figure 4 shows the ROC AUC curves on the validation dataset of the corresponding models. We evaluate three different weighting strategies: proportional weighting (Eq. 1) with similarity functions based on morgan (circular) fingerprints and hyperbolic embeddings, and window weighting (Eq. 3) based on molecular descriptors. Interestingly, it could be seen that among the all edge weighting (Fig. 4a) and node initialization (Fig. 4b) strategies, only one of each shows improved performance over the base model. In particular, window weighting based on molecular descriptors and node initialization with morgan (circular) fingerprints work best.

Table 2 shows the ratio of false positives (FPR) and false negatives (FNR) for different modifications of the SimVec model. It could be seen that in most cases FPR is higher than FNR, meaning that a model tends to predict the existence of nonexistent edges more frequently than absence of existing edges. However, for SimVec_chem and SimVec_chem_weighted models FNR is higher than FPR.

### Impact of different modifications

It is clear from the results in Table 1 that the use of loss based on single side effects increases the model performance the most. This finding could be explained by the fact that the information about individual side effects is most relevant to the information about polypharmacy side effects.

Then the use of weighted similarity edges improves the results by allowing the model to propagate information between loosely connected nodes.

Structure-aware initialization makes the smallest contribution to performance boost . There are two possible explanations .

First, in our study we do not perform chemical feature selection, thus many of the used features are potentially redundant, while increasing the node embedding size. This could potentially reduce performance. That is, deep investigation and selection of appropriate chemical features is one of the approaches to the incorporation of chemical information into KG-based models.

Second, it could be the case that the chemical information itself is not a good source of prior information for the prediction of polypharmacy side effects. In that case, it is important to use other types of features as the main source of prior information and incorporate chemical structures as additional properties. This is what is happening in the final SimVec_full model, in which chemical information is used along with single side effects. Some of the other suitable properties can be changes in gene expression and morphological alterations.

### Negative sampling strategies

On top of SimVec_SE_weighted_ model , as the simplest model with the most significant improvement, we conducted experiments with different negative sampling strategies. Table 3 shows the results of the evaluation of six proposed alternatives to uniform negative sampling, described in the Methods section. It could be seen that the Stay Positive method shows the best results, while the Exploration, NSCaching, and Strong NSCaching models show similar accuracy with respect to standard deviations.

**Fig. 5:**
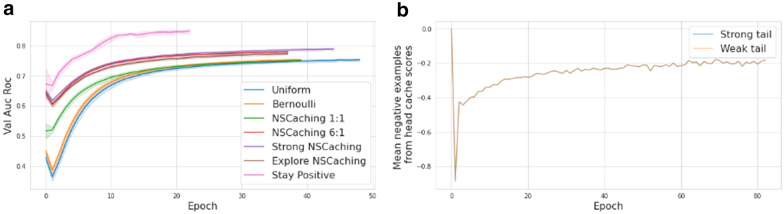
Negative sampling models. **a** In terms of validation ROC AUC, Stay Positive model performs significantly better then others. Other models show similar accuracy, albeit Strong NSCAching, NSCaching 6:1 and Expolre NSCaching work slightly better then NSCaching 1:1, Bernoulli and Uniform sampling. **b** Scores of negative examples, sampled from weak and strong tail caches, are indistinguishable. That is, sampling from strong neighbour cache does not introduce meaningful improvement

The same behavior could be seen during model training on the validation data set (Fig. 5a). In order to investigate the reasons for these findings, we examined the cached scores of negative examples for weak and strong nodes. If the scores of negative examples for strong nodes were higher than for weak nodes, then the Strong NSCaching method would be able to show better performance. However, Fig. 5b shows that there is no difference in scores for both types of nodes, which means that the weak nodes do not benefit from sampling from their strong neighbors. These results show that a slight improvement of Strong NSCaching might be due to chance, and both Exploration and NSCaching_6:1_ work well for the current task.

We then test SimVec_SE_weighted_ with Stay Positive and cache-based sampling on the uniform split. Although Stay Positive works best on weak nodes split, it shows ROC AUC 0.945 and AUC PR 0.915 on the uniform one, which is worse than the original TriVec model’s accuracy (ROC AUC 0.975, AUC PR 0.966). In contrast, with cache-based sampling , our models show slightly better performance than TriVec, namely, ROC AUC 0.976 and AUC PR 0.967. As a result, we suggest using cache-based sampling strategies as a more stable solution.

Our final model, SimVec_full_ with NSCaching_6:1_ shows ROC AUC 0.79 and AUC PR 0.76 on weak nodes split, and ROC AUC 0.975 and AUC PR 0.955 on uniform nodes split.

### Computational resources and end-user usage

Table 4 shows the computational resources consumed by the models during the training on a server with one Tesla k80 Accelerator, 61 GiB memory and 4 virtual CPUs. All of the modifications of SimVec models are fast learning and lightweight in terms of resource consumption, and can be trained on a CPU server. Table 5 shows the training time of different SimVec modifications on a server with 4 virtual CPUs and 61 GiB memory.

One of the most important metrics for an end-user is prediction time. For all the SimVec modifications, it takes about 2 min on a server with GPU acceleration and 10 minutes on a server without GPU acceleration to predict polypharmacy side effects for 20,000 pairs of drugs.

### Opportunities and limitations

The SimVec model allows predicting polypharmacy side effects for new drugs, the interactions of which are little known. Given a new drug with unknown polypharmacy assertions, the SimVec model can predict the presence of a certain polypharmacy side effect with another drug.

The model learns quickly compared to other state-of-the-art models and consumes a similar amount of GPU and CPU resources (Table 4). It makes it possible to retrain the model on different datasets.

The main limitation of our work is the restricted application domain and limited scalability. With an increase in the number of drug nodes, the number of required weighted similarity edges increases significantly.

## Conclusion

This paper examines the weak nodes problem in KG-based polypharmacy side effects prediction models and significantly improves predictions for such drugs with limited known polypharmacy assertions. The weak nodes in the knowledge graph represent new drugs that should be analyzed with special attention. Our work shows that current state-of-the-art approaches in the field struggle with this scenario. Our proposed model, SimVec, significantly outperforms other KG-based models and shows the ability to work efficiently with new drugs.

It seems reasonable to extrapolate predictions from a pair of drugs to variable-sized sets of drugs in future work. In practical terms, there is a clear need for a general inductive model for the polypharmacy task.

## Data Availability

The preprocessed dataset, provided within the Decagon paper [4] and used in this paper, can be downloaded from the Stanford Network Analysis Project’s website (http://snap.stanford.edu/decagon/). The open source implementation of the SimVec model, including preprocessed data to reproduce results, is available at GitHub (https://github.com/jbr-ai-labs/simvec).

## Appendix

The list of used molecular descriptor IDs:

MaxEStateIndex, MinEStateIndex, MaxAbsEStateIndex, MinAbsEStateIndex, MolWt, HeavyAtomMolWt, ExactMolWt, NumValenceElectrons, NumRadicalElectrons, MaxPartialCharge, MinPartialCharge, MaxAbsPartialCharge, MinAbsPartialCharge, FpDensityMorgan1, FpDensityMorgan2, FpDensityMorgan3, BalabanJ, BertzCT, Chi0, Chi0n, Chi0v, Chi1, Chi1n, Chi1v, Chi2n, Chi2v, Chi3n, Chi3v, Chi4n, Chi4v, HallKierAlpha, Ipc, Kappa1, Kappa2, Kappa3, LabuteASA, PEOE_VSA1, PEOE_VSA10, PEOE_VSA11, PEOE_VSA12, PEOE_VSA13PEOE_VSA14, PEOE_VSA2, PEOE_VSA3, PEOE_VSA4, PEOE_VSA5, PEOE_VSA6, PEOE_VSA7, PEOE_VSA8, PEOE_VSA9, SMR_VSA1, SMR_VSA10, SMR_VSA2, SMR_VSA3, SMR_VSA4, SMR_VSA5, SMR_VSA6, SMR_VSA7, SMR_VSA9, SlogP_VSA1, SlogP_VSA10, SlogP_VSA11, SlogP_VSA12, SlogP_VSA2, SlogP_VSA3, SlogP_VSA4, SlogP_VSA5, SlogP_VSA6, SlogP_VSA7, SlogP_VSA8, TPSA, EState_VSA1, EState_VSA10, EState_VSA11, EState_VSA2, EState_VSA3, EState_VSA4, EState_VSA5, EState_VSA6, EState_VSA7, EState_VSA8, EState_VSA9, VSA_EState1, VSA_EState10, VSA_EState2, VSA_EState3, VSA_EState4, VSA_EState5, VSA_EState6, VSA_EState7, VSA_EState8, VSA_EState9, FractionCSP3, HeavyAtomCount, NHOHCount, fr_hdrzine, fr_methoxy, fr_morpholine, fr_unbrch_alkane, fr_urea.

## Abbreviations

KG: Knowledge graph
